# Changing trends in liver transplantation indications in Saudi Arabia: from hepatitis C virus infection to nonalcoholic fatty liver disease

**DOI:** 10.1186/s12876-021-01828-z

**Published:** 2021-06-01

**Authors:** Saleh A. Alqahtani, Dieter C. Broering, Saad A. Alghamdi, Khalid I. Bzeizi, Noara Alhusseini, Saleh I. Alabbad, Ali Albenmousa, Nasreen Alfaris, Faisal Abaalkhail, Waleed K. Al-hamoudi

**Affiliations:** 1 Liver Transplant Center, King Faisal Specialist Hospital & Research Center, Riyadh, Saudi Arabia; 2 Division of Gastroenterology and Hepatology, Johns Hopkins University, Baltimore, MD USA; 3 College of Medicine, Alfaisal University, Riyadh, Saudi Arabia; 4 Obesity, Endocrine, and Metabolism Center, King Fahd Medical City, Riyadh, Saudi Arabia; 5 Department of Medicine, Gastroenterology Section, King Faisal Specialist Hospital and Research Center, Riyadh, Saudi Arabia; 6 Liver Disease Research Center, College of Medicine, King Saud University, Riyadh, Saudi Arabia

**Keywords:** Liver disease, Liver transplantation, Nonalcoholic steatohepatitis, Saudi Arabia, Trend analysis

## Abstract

**Background:**

Several trend analyses on liver transplantation (LT) indications have been published in the U.S. and in other countries, but there are limited data on LT indication trends in Saudi Arabia (SA), especially since the availability of direct-acting antivirals (DAAs) treatment for hepatitis C virus (HCV). This study aimed to analyze trends in the frequency of LT indications among LT recipients in SA over a 19-year period and examine associations between etiologic-specific trends and clinicodemographic characteristics.

**Methods:**

This retrospective study analyzed clinical and surgical data of adult patients (n = 1009) who underwent LT at the King Faisal Specialist Hospital & Research Center (Riyadh, SA) between 2001 and 2019. Spearman’s rank correlation, Poisson regression, and Joinpoint regression analysis were employed to assess changes in LT etiologic trends.

**Results:**

In the first period (2001–2010), the main LT indications were HCV (41.9%) and hepatitis B virus (HBV) (21.1%), but nonalcoholic steatohepatitis (NASH) (29.7%) surpassed HCV (23.7%) as the leading LT indication in the second period (2011–2019); and the trends were significant in correlation analyses [incidence rate ratio (IRR) = 1.09 (1.06–1.13) for NASH; IRR = 0.93 (0.91–0.95) for HCV]. In the Joinpoint regression analysis, increases in NASH from 2006 to 2012 (+ 32.1%) were statistically significant, as were the decreases in HCV from 2004 to 2007 (− 19.6%) and from 2010 to 2019 (− 12.1%). Similar patterns were observed in LT etiological comparisons before and after the availability of DAAs and within hepatocellular carcinoma stratifications.

**Conclusions:**

Trends in the epidemiology of LT indications among LT recipients in SA have changed over a 19-year period. Most notably, NASH has eclipsed HCV in the country due to the effective treatment strategies for HCV. These trends in NASH now need an aggressive public health response to minimize and avert future onset of additional clinical and economic strains on health care systems and LT centers in SA.

## Background

Worldwide, chronic liver disease (CLD) is estimated to cause 2 million annual deaths [1]. Among those who die from CLD, half die from cirrhosis-associated complications, and the other half die from viral hepatitis (VH) or hepatocellular carcinoma (HCC). Currently, liver transplantation (LT) is the only curative option for patients living with end-stage liver disease (ESLD) [2]. Before 2014, chronic hepatitis C virus (HCV) infection was the most common indication for non-HCC LT listing in the United States (U.S.) [3]. The advent of direct-acting antivirals (DAAs) has revolutionized treatment for HCV, reduced progression to ESLD in a significant number of patients living with HCV, and lessened the need for LT in patients living with HCV [4, 5]. As a result, HCV was replaced by alcoholic liver disease (ALD) in 2015 as the primary listing indication for LT in the U.S. (19.7% vs. 28.5% of non-HCC LT listing, respectively) [3]. Interestingly, NASH and ALD continue to increase in prevalence among LT candidates, with NASH and ALD accounting for 28.1% and 37.7% of the total number of listings without HCC in 2019, which were 5.4 times and 2.2 times higher than the 2002 values, respectively [3]. Similar trends have been observed in Europe. The 2018 Annual Report of the European LT Registry noted a sharp decline in VH-related indications over the last 50 years, and the report projected that NASH will be the leading indication for LT in Europe within the next decade [6].

NASH is the most severe form of nonalcoholic fatty liver disease (NAFLD) [7]. The main risk factors for NAFLD are obesity, type 2 diabetes mellitus (T2DM), dyslipidemia, and insulin resistance [8]. As the worldwide prevalence of obesity has tripled from 1975 to 2016 [9], the current obesity pandemic will likely accelerate worldwide increases in the prevalence of NAFLD, and consequently, NASH [10]. Such an undesired hike in prevalence may cause a substantial rise in liver-related complications and escalate the need for LT [11]. Sex dimorphism is reported in the NAFLD prevalence and associated outcomes, highlighting the need for detailed investigations of sex differences in these patients [12]. Additionally, NAFLD is found to cause fatal and non-serious cardiovascular disease (CVD) and its associated adverse outcomes [13]. Patients with liver cirrhosis develop pre-LT electrocardiographic abnormalities with symptoms like lengthened QTc interval and ST depression; they are 14-times more likely to develop a post-LT CVD event [14]. The odds of developing serious CVD events are reportedly high in patients with severe NAFLD, prompting clinicians to consider NAFLD as an independent risk factor for CVD events [15–17]. Biomarkers of liver fibrosis correlate CVD risk scores in patients with biopsy-confirmed CLD, stressing the need for continuous testing of liver disease status while attempting to stratify CVD events [18]. Likewise, co-screening for T2DM in NAFLD patients is also recommended, which would allow for early interventions [19]. A registry-based study found that LT recipients for NASH pose a higher risk of developing de novo T2DM, indicating the presence of pre-existing metabolic syndrome alongside NASH [20].

In Saudi Arabia (SA), the prevalence of obesity is approximately 38%, and it is projected to reach 45% by 2025 [9]. Given the high prevalence of obesity in the Middle East and that other countries have observed rapid changes in LT indications, there is a need to examine current trends in LT indications in SA. Cirrhosis due to sclerosing cholangitis was the indication for the first-ever LT in SA [21], but since then, there have been dynamic changes in LT indications in the country. Several trend analyses on LT indications have been published in the U.S. and other countries [6, 22–24], but there are limited data on LT indication trends in the Middle East, especially after 2014, when the DAAs became available in SA for treating HCV [25]. It is important to characterize and to stay abreast of probable changes in LT indication trends to mobilize and support public health and health policy resources for optimal reduction and management of disease burden and the associated complications in the context of LT. In this retrospective study, we analyzed trends in the frequency of LT indications among LT recipients in a single-center in SA over a 19-year period and examined associations between etiologic-specific trends and clinicodemographic characteristics.

## Methods

### Study design

In this single-center, retrospective study, we collected and analyzed anonymized clinical and surgical data of adult patients who underwent LT at the King Faisal Specialist Hospital & Research Center (KFSHRC, Riyadh, SA) between 2001 and 2019. All adult patients who underwent LT (n = 1009), irrespective of etiology, were included in the analysis.

### Outcomes of interest

Primary outcomes of interest were (1) the most frequent LT indications over the full study period (2001 to 2019) and between two sub-periods (2001 to 2010 vs. 2011 to 2019), (2) year-on-year trends of the most frequent LT indications, and (3) ways in which the two sub-periods and pre- and post-DAA eras (pre-2014 vs. 2014 to 2019, DAAs were not widely available in SA until 2014) differed in patient characteristics and LT indications, respectively. Secondary outcomes of interest were the most frequent LT indications among patients with and without HCC, and year-on-year trends of the most frequent LT indications stratified by HCC status. Indications were extracted from electronic medical records and grouped into 8 categories: NASH, HCV, hepatitis B virus (HBV), Wilson’s disease, primary sclerosing cholangitis (PSC), autoimmune hepatitis (AIH), schistosomiasis (SCH), and other (i.e., “other” was a composite variable created to group infrequent indications (< 2% of all LTs).

### Variables

The following clinicodemographic characteristics were extracted from electronic medical records of LT recipients from 2001 to 2019: age, sex, body mass index (BMI), waitlist times, donor sources, Model for End-Stage Liver Disease (MELD) scores, and HCC status.

### Statistical analysis

Categorical variables were presented as frequencies and proportions and continuous variables as mean ± standard deviation or median and interquartile range as appropriate. The Kruskal–Wallis test and the Pearson χ^2^ test were used to compare two periods (2001 to 2010 vs. 2011 to 2019) and patients with and without HCC on continuous variables and categorical variables. The following tests were employed to assess changes in LT etiological trends across the full study period (2001 to 2019), between pre- and post-DAA eras in SA (pre-2014 vs. 2014–2019), and by HCC status: Spearman’s rank correlation (percentage of patients by year for each etiology), Poisson regression (the Pearson χ^2^ goodness-of-fit test was used to assess model fit, incidence rate ratios [IRR] presented) and Joinpoint regression analysis (for exploration of any periods within the study period with significant annual percentage changes). All statistical analyses were performed using Stata (version 14, Stata Corp, College Station, Texas, U.S.) apart from Joinpoint regression analysis, which was performed using the Joinpoint Regression Program.

### Ethical approval and informed consent

On December 3, 2019, the Institutional Review Board of KFSHRC (Riyadh, SA) approved this study and waived the need for informed consent due to the retrospective and anonymous nature of the study (Approval Number: RAC 2,171,177). This study was carried out in accordance with the World Medical Association (WMA) Declaration of Helsinki (adopted: 1964; last amended: 2013) and the WMA statement on measures for the prevention and fight against transplant-related crimes (adopted: October 2020).

## Results

### Demographic and clinical characteristics of LT recipients

Among those who underwent LT between 2001 and 2019 (Table 1), the median age was 55 (45–62) years, the median BMI was 26.1 (22.9–30.5), and the majority were male (62.8%). The most prevalent LT etiologies were HCV (28.4%), NASH (24.6%), and HBV (22.2%).

**Table 1:** Characteristics of LT recipients, overall and by major etiology

	Overall	NASH	HCV	HBV	Wilson's disease	PSC	AIH	SCH	Other
N transplants	1009	248 (24.6%)	287 (28.4%)	224 (22.2%)	23 (2.3%)	28 (2.8%)	89 (8.8%)	21 (2.1%)	89 (8.8%)
Age [Median (IQR)]	55 (45–62)	59 (53–64)	58 (52–64)	54 (48–61)	23 (19–29)	40 (32–47)	32 (23–45)	60 (57–64)	34 (25–54)
Male [n (%)]	634 (62.8%)	174 (70.2%)	162 (56.5%)	167 (74.6%)	12 (52.2%)	13 (46.4%)	38 (42.7%)	21 (100.0%)	47 (52.8%)
BMI [Median]	26.1 (22.9–30.5)	26.6 (23.6–32.0)	26.9 (23.9–30.8)	27.3 (23.4–31.3)	22.8 (20.7–24.3)	23.5 (20.1–27.2)	24.2 (21.6–28.2)	25.9 (23.0–29.3)	23.6 (20.1–27.3)
*BMI group* [n (%)]									
Underweight	49 (4.9%)	6 (2.4%)	7 (2.4%)	6 (2.7%)	4 (17.4%)	4 (14.3%)	7 (7.9%)	0 (0.0%)	15 (16.9%)
Normal weight	366 (36.3%)	78 (31.5%)	90 (31.4%)	73 (32.6%)	13 (56.5%)	14 (50.0%)	45 (50.6%)	10 (47.6%)	43 (48.3%)
Pre-obesity	308 (30.5%)	84 (33.9%)	99 (34.5%)	69 (30.8%)	3 (13.0%)	8 (28.6%)	20 (22.5%)	7 (33.3%)	18 (20.2%)
Obesity (class 1)	194 (19.2%)	52 (21.0%)	62 (21.6%)	53 (23.7%)	1 (4.4%)	1 (3.6%)	13 (14.6%)	4 (19.1%)	8 (9.0%)
Obesity (class 2)	60 (6.0%)	15 (6.1%)	19 (6.6%)	18 (8.0%)	1 (4.4%)	0 (0.0%)	4 (4.5%)	0 (0.0%)	3 (3.4%)
Obesity (class 3)	32 (3.2%)	13 (5.2%)	10 (3.5%)	5 (2.2%)	1 (4.4%)	1 (3.6%)	0 (0.0%)	0 (0.0%)	2 (2.3%)
Waitlist time [Median days]	49 (15–139)	42 (20–103)	58 (15–160)	49 (15–141)	75 (10–110)	64 (24–153)	36 (14–156)	102 (42–131)	49 (15–155)
LT from living donor [n (%)]	628 (62.2%)	179 (72.2%)	159 (55.4%)	150 (67.0%)	10 (43.5%)	19 (67.9%)	47 (52.8%)	16 (76.2%)	48 (53.9%)
MELD score [Median]	22 (16–25)	22 (17–24)	20 (15–22)	22 (17–25)	30 (17–37)	21 (16–26)	24 (19–31)	20 (17–22)	24 (18–30)
HCC candidate [n (%)]	282 (28.0%)	59 (23.8%)	123 (42.9%)	87 (38.8%)	1 (4.4%)	0 (0.0%)	2 (2.3%)	4 (19.1%)	6 (6.7%)

LT, liver transplant; NASH, nonalcoholic steatohepatitis; HCV, hepatitis C virus; HBV, hepatitis B virus; PSC, primary sclerosing cholangitis; AIH, autoimmune hepatitis; SCH, schistosomiasis; IQR, interquartile range; BMI, body mass index; MELD, model for end-stage liver disease; HCC, hepatocellular carcinoma

### Trends in LT etiology

**Fig. 1:**
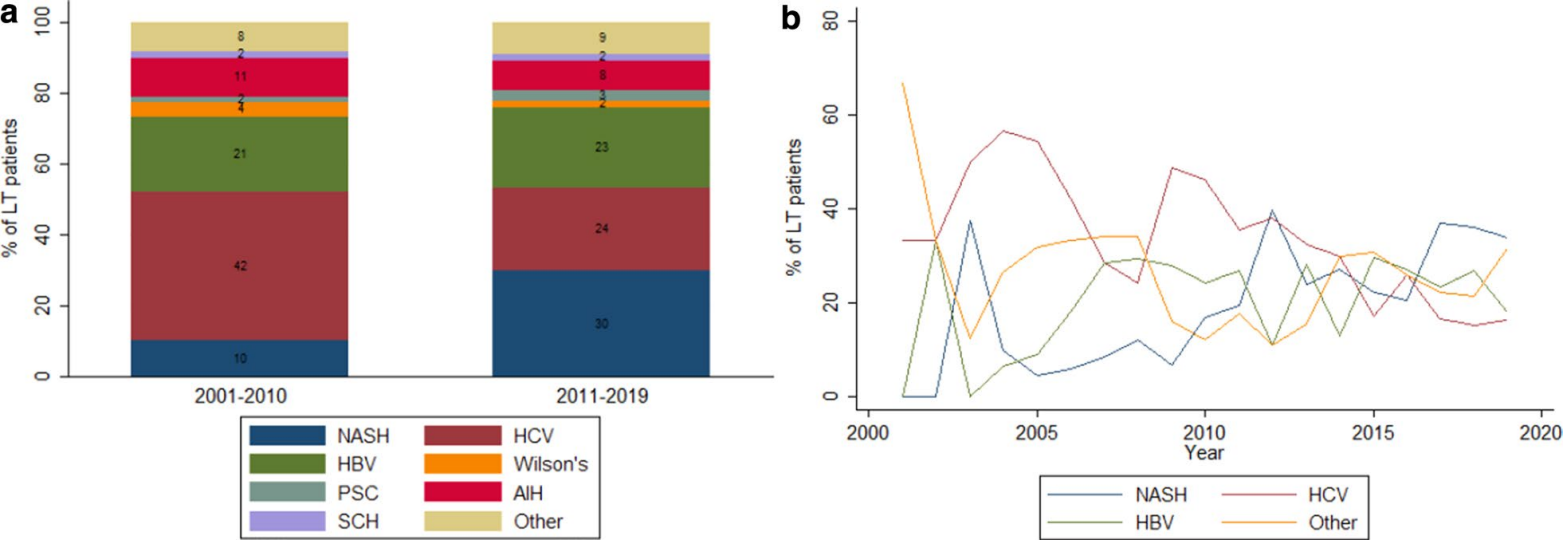
**a** Distribution of etiologies by period: 2001–2010 and 2011–2019. **b** Percentage distribution of etiologies by year: 2001–2019

In the first period (2001–2010), the main LT indications were HCV (41.9%) and HBV (21.1%) (Fig. 1a). In the second period (2011–2019), NASH (29.7%) surpassed HCV (23.7%) as the leading LT indication, while other indications remained relatively stable in both periods. In addition, since 2016, NASH has remained the leading LT indication (Fig. 1b). Across the full study period (2001–2019) (Table 2), the decline in HCV and increase in NASH as the leading LT etiology was statistically significant in correlation analyses (IRR = 1.09 [1.06–1.13] for NASH; IRR = 0.93 [0.91–0.95] for HCV). In the Joinpoint regression analysis, increases in NASH from 2006 to 2012 (32.1% increase) were statistically significant, and decreases in HCV from 2004 to 2007 (− 19.6%) and from 2010 to 2019 (− 12.1%) were statistically significant.

**Table 2:** Year-on-year trend analyses for LT etiology: 2001–2019

	NASH	HCV	HBV	Other
*Spearman rank correlation*				
*Ρ*	0.68	− 0.71	0.24	− 0.34
*p* value	0.001	< 0.001	0.318	0.154
*Joinpoint regression*				
	*p* < 0.05	*p* < 0.05	*p* < 0.05	Not significant
	2006–2012 APC: +32.1%	2004–2007 APC: − 19.6%	2007–2019 APC: − 1.6%	
		2010–2019 APC: − 12.1%		
*Incidence rate ratio*				
IRR	1.09	0.93	1.02	1.00
95% CI	1.06–1.13	0.91–0.95	0.99–1.05	0.97–1.03
*p* value	< 0.001	< 0.001	0.287	0.906

### Trends in patient characteristics

Compared to patients who underwent LT in the first period (Table 3), patients in the second period were older (median age, 50 vs. 56), had higher median MELD scores (median, 19 vs. 22), longer waitlist times (median, 43 vs. 82 days), and were more likely to receive LT from a living donor (27.9% vs. 74.5%) and to have HCC (21.5% vs. 30.2%).

**Table 3:** Characteristics of LT recipients by period: 2001–2010 and 2011–2019

	2001–2010	2011–2019	*p* value
N transplants	265	744	
LT etiology*^†^			
NASH	27 (10.2%)	221 (29.7%)	< 0.001
HCV	111 (41.9%)	176 (23.7%)	
HBV	56 (21.1%)	168 (22.6%)	
Wilson's disease	11 (4.2%)	12 (1.6%)	
PSC	4 (1.5%)	24 (3.2%)	
AIH	29 (10.9%)	60 (8.1%)	
SCH	5 (1.9%)	16 (2.2%)	
Other	22 (8.3%)	67 (9.0%)	
Age [Median (IQR)]*^‡^	50 (37–57)	56 (47–63)	< 0.001
Male†	166 (62.6%)	468 (62.9%)	0.940
BMI [Median (IQR)]^‡^	26.0 (22.4–30.0)	26.2 (22.9–30.6)	0.299
*BMI group* [n (%)]^†^			
Underweight	13 (4.9%)	36 (4.8%)	0.767
Normal weight	97 (36.6%)	269 (36.2%)	
Pre-obesity	81 (30.6%)	227 (30.5%)	
Obesity (class 1)	47 (17.7%)	147 (19.8%)	
Obesity (class 2)	15 (5.7%)	45 (6.1%)	
Obesity (class 3)	12 (4.5%)	20 (2.7%)	
Waitlist time [Median days (IQR)]*^‡^	82 (21–206)	43 (15–112)	< 0.001
LT from living donor [n (%)]*^†^	74 (27.9%)	554 (74.5%)	< 0.001
MELD score [Median (IQR)]*^‡^	19 (14–28)	22 (17–25)	0.001
HCC candidate [n (%)]*^†^	57 (21.5%)	225 (30.2%)	0.007

LT, liver transplant; NASH, nonalcoholic steatohepatitis; HCV, hepatitis C virus; HBV, hepatitis B virus; PSC, primary sclerosing cholangitis; AIH, autoimmune hepatitis; SCH, schistosomiasis; BMI, body mass index; MELD, model for end-stage liver disease; HCC, hepatocellular carcinoma

^*^Significant difference between 2001–2010 and 2011–2019

^†^Periods compared with χ^2^ test

^‡^Periods compared with Kruskal–Wallis equality of populations rank test

### Trends in LT etiology before and after the availability of DAAs

**Fig. 2:**
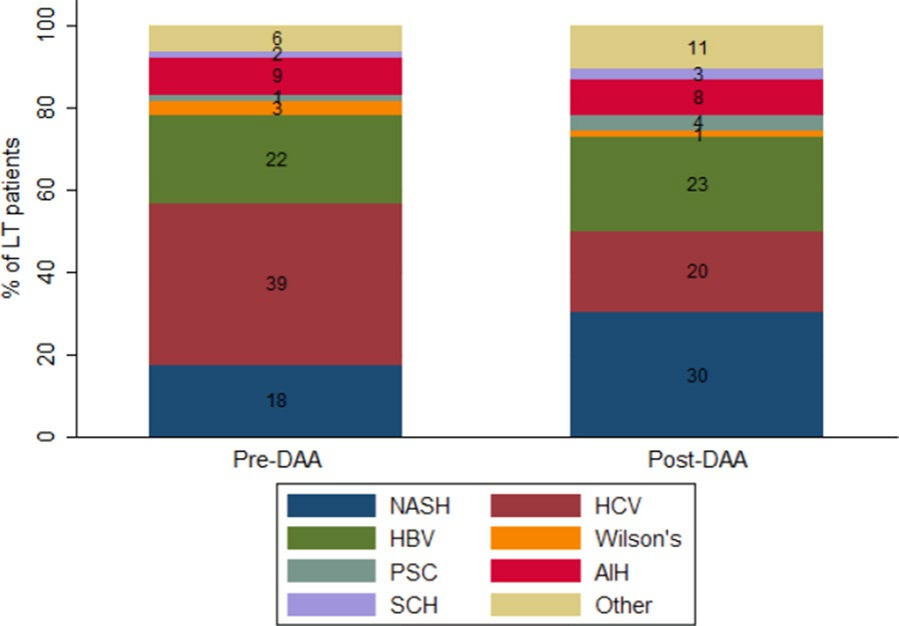
Distribution of LT etiologies before (pre-2014) and after (2014–2019) the availability of direct acting antivirals (DAAs)

During the pre-DAA era (i.e., pre-2014, before the availability of DAAs in SA) (Fig. 2), HCV (39%) was the leading LT etiology, followed by HBV (22%). However, in the post-DAA era (i.e., 2014–2019, during the availability of DAAs in SA), NASH emerged as the leading LT etiology, followed by HBV (23%). Comparing LT etiologies before and after the availability of DAAs in SA (Table 4), increases in NASH were statistically significant (17.6% to 30.3%, IRR = 1.72 [1.32–2.25]), as well as the decreases in HCV (39.1% to 19.7%, IRR = 0.50 [0.40–0.64]). There were no differences in statistical significance between periods for other etiologies.

**Table 4:** Distribution of primary LT etiologies before and after the availability of direct-acting antivirals

	NASH	HCV	HBV	Other
Pre-DAA	80 (17.6%)	178 (39.1%)	98 (21.5%)	99 (21.8%)
Post-DAA	168 (30.3%)	109 (19.7%)	126 (22.7%)	151 (27.3%)
*IRR*				
IRR	1.72	0.50	1.06	1.25
95% CI	1.32–2.25	0.40–0.64	0.81–1.37	0.97–1.61
*p* value	< 0.001	< 0.001	0.686	0.081

IRR compares pre- and post-DAA periods

LT, liver transplant; NASH, Nonalcoholic steatohepatitis; HCV, Hepatitis C virus; HBV, Hepatitis B virus; DAA, Direct-acting antiviral; IRR, incidence rate ratio; CI, confidence interval

### Trends in LT etiology by HCC status

**Fig. 3:**
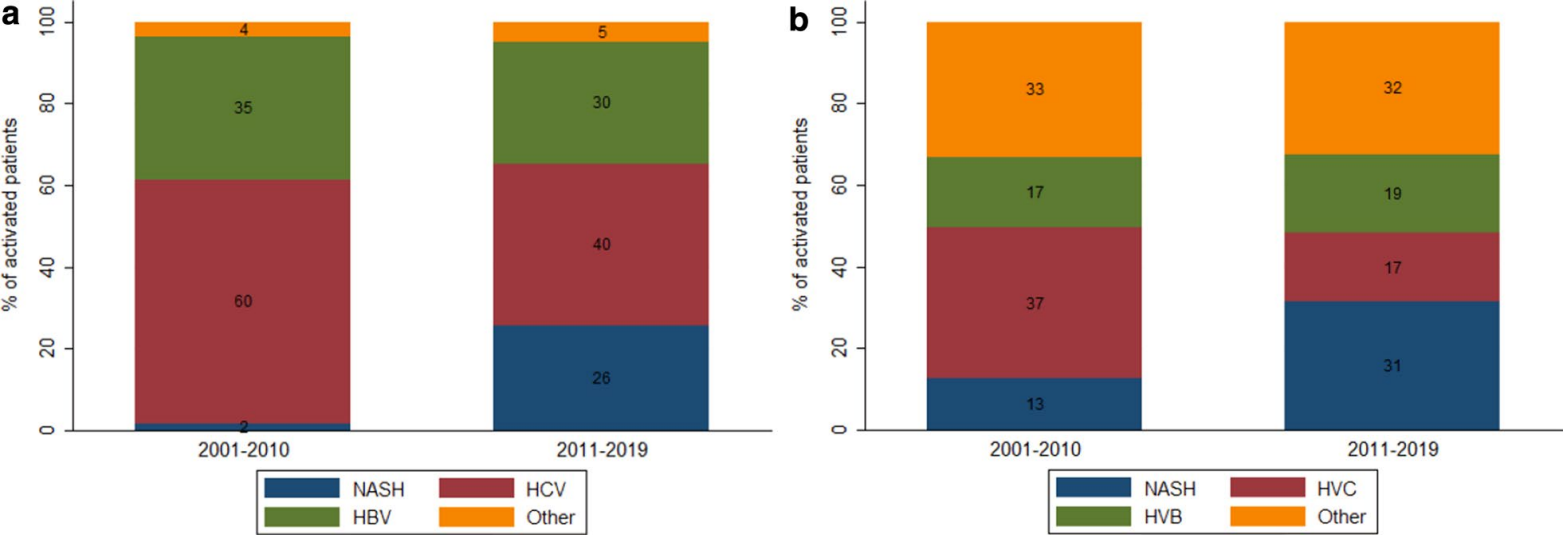
Distribution of etiologies among recipients during 2001–2010 and 2011–2019 - **a**) With HCC. **b**) Without HCC

Compared to patients without HCC (Table 5), patients with HCC were more likely to be male (60.0% vs. 70.2%) and older (median, 53 vs. 60), have higher BMI (median, 25.7 vs. 26.6) and MELD (median, 21 vs. 22) scores. Among patients with HCC (Fig. 3a), HCV and HBV were the leading LT etiologies for both periods (2001–2010: 60% and 35%; 2011–2019: 40% and 30%, respectively). Similarly, HCV and HBV were the leading LT etiologies among patients without HCC between 2001 and 2010 (Fig. 3b), but NASH (31%) emerged as a leading LT etiology between 2011 and 2019. Stratifying patients with and without HCC within LT etiologies (Table 6), increases in NASH among patients with and without HCC were statistically significant (IRR = 1.16 [1.07–1.26]; IRR = 1.08 [1.04–1.12], respectively), and decreases in HCV among patients with and without HCC were statistically significant over the study period (IRR = 0.94 [0.90–0.98]; IRR = 0.91 [0.89–0.94], respectively).

**Table 5:** Characteristics of the LT recipients with and without HCC

	With HCC	Without HCC	*p* value
*N transplants*	282	727	
Age [Median (IQR)]	60 (54–64)	53 (38–60)	< 0.001
Male [n (%)]	198 (70.2%)	436 (60.0%)	0.003
BMI [Median (IQR)]	26.6 (24.1–30.5)	25.7 (22.6–30.5)	0.018
*BMI group* [n (%)]			
Underweight	6 (2.1%)	43 (5.9%)	0.005
Normal weight	87 (30.9%)	279 (38.4%)	
Pre-obesity	107 (37.9%)	201 (27.7%)	
Obesity (class 1)	56 (19.9%)	138 (19.0%)	
Obesity (class 2)	18 (6.4%)	42 (5.8%)	
Obesity (class 3)	8 (2.8%)	24 (3.3%)	
Waitlist time [Median days (IQR)]	49 (15–151)	49 (16–134)	0.9038
LT from living donor [n (%)]	183 (64.9%)	445 (61.2%)	0.279
MELD score [Median (IQR)]	22 (15–22)	21 (16–27)	< 0.001

LT, liver transplant; HCC, hepatocellular carcinoma; IQR, interquartile range; BMI, Body mass index; MELD, model for end-stage liver disease

**Table 6:** Year-on-year trend analyses of LT etiologies by HCC status: 2001–2019

	NASH		HCV		HBV		Other	
	With HCC	Without HCC	With HCC	Without HCC	With HCC	Without HCC	With HCC	Without HCC
*Spearman rank correlation*								
*ρ*	0.66	0.75	− 0.51	− 0.73	0.25	0.43	0.61	− 0.11
*p* value	0.002	0.000	0.025	0.000	0.300	0.069	0.005	0.652
*Joinpoint regression*								
	*p* < 0.05*	*p* < 0.05	*p* < 0.05*	*p* < 0.05	NS	NS	NS	NS
	2011–2019 APC: 10.4%	2004–2012 APC: 24.5%	2006–2019 APC: − 4.9%	2010–2019 APC: − 12.5%				
*Incidence rate ratio*								
IRR	1.16	1.08	0.94	0.91	1.00	1.02	1.12	1.00
95% CI	1.07–1.26	1.04–1.12	0.90–0.98	0.89–0.94	0.95–1.06	0.98–1.05	0.95–1.31	0.98–1.03
*p* value	< 0.001	< 0.001	0.002	< 0.001	0.873	0.414	0.171	0.768

LT, liver transplant; HCC, hepatocellular carcinoma; NASH, nonalcoholic steatohepatitis; HCV, hepatitis C virus; HBV, hepatitis B virus; APC, annual percentage change; IRR, incidence rate ratio; CI, confidence interval; NS, not significant

## Discussion

We retrospectively analyzed trends in the frequency of LT indications among LT recipients in a single-center in SA over a 19-year period and examined associations between etiologic-specific trends and clinicodemographic characteristics. Several notable findings materialized from the analysis. First, NASH surpassed HCV as the leading LT indication among LT recipients in SA. In particular, NASH surpassed HCV for the first time in 2012; HCV regained its place for four years until 2016 when NASH outpaced HCV again and has remained the leading LT indication among LT recipients in SA. Second, declines in HCV and increases in NASH as leading etiologies over the study period were significant in correlation analyses. This trend is consistent with a recently published LT trend analysis of a large retrospective cohort in the U.S [3]. The study determined that NASH was the fastest increasing indication from 2002 to 2019, including among patients with and without HCC, and NASH surpassed HCV and emerged as the second leading indication only after ALD in the U.S. Although ALD was ahead of NASH in the U.S., it is less frequent in SA due to socioreligious taboos and legal bans on the sale and consumption of alcohol. Nevertheless, rapid changes in NASH trends in the last two decades are unprecedented in both SA and the U.S.

Given this shift in epidemiologic trends, perhaps it is now time to consider prioritization of NASH and start applying lessons and best practices learned from HCV to NASH (i.e., public health awareness campaigns; government-supported policy recommendations—to support and facilitate early screening, identification, and linkage to care; efforts to increase access to care; and pharmacologic and staging advancements). In response to increasing changes in the epidemiology of HCV, not long-ago, international health agencies and countries heavily burdened with HCV responsively and purposefully dedicated clinical and economic resources to combat and control HCV prevalence [26]. Likewise, efforts to prioritize and highlight the growing burden of NASH have the potential to downturn the prevalence and incidence of LT waitlisting, similar to recent downturns in the prevalence and incidence of HCV-associated morbidity and mortality in SA and other countries abroad.

Third, the epidemiology of LT etiology trends among LT recipients changed after the availability of DAAs in SA in 2014. In pre- and post-DAA era comparisons, there were significant year-on-year increases in NASH and significant year-on-year decreases in HCV among LT recipients, and NASH ultimately eclipsed HCV in the current DAA era in SA. These respective findings are consistent with pre- and post-DAA LT etiology trends in Europe and the U.S [5, 27]. Though NASH is not the leading LT etiology among LT recipients in the current DAA era in the U.S., NASH is the leading etiology for waitlisted LT patients, it is only second to HCV among LT recipients, and is steadily rising year upon year; whereas, HCV is declining year upon year due to effective treatment strategies, and it is quite possible that NASH in the current DAA era will overtake HCV as the leading indication in the U.S. in the coming years [27, 28].

Given the remarkable advancements and benefits of pangenotypic DAAs for HCV treatment, this changing trend was somewhat expected in the current study. Not only are the majority of HCV patients (> 95%) able to achieve virologic cure (i.e., sustained virological response [SVR]) with DAAs, but a large majority of HCV patients who achieve SVR are able to achieve cirrhosis regression [29, 30], and in some instances, resolve manifestations of hepatic decompensation [31, 32]. In turn, DAAs have reduced the number of patients who normally would have been waitlisted due to HCV-associated CLD in the pre-DAA era, and DAAs have delisted an unprecedented number of LT waitlisted patients. Although a desirable public health and clinical outcome, an unintended consequence of the emergence and wide usage of DAAs has been the emergence of NASH as a leading LT etiology. It is probable that this trend will continue to balloon in the coming decades because despite the various clinical trials testing compounds for NASH therapy [33], there are no pharmacologic treatments for NASH that are as highly efficacious and effective as DAAs for HCV. Accordingly, healthcare systems and LT centers in SA and elsewhere worldwide are strongly encouraged to consider pre-emptive and novel ways to prevent, manage, and reduce NASH-associated CLD complications.

Lastly, in SA, NASH had an upward trend, while HCV showed a downward trend in patients with and without HCC, respectively. This finding was identical to findings in the U.S., as NASH was the most rapidly increasing indication for LT in the U.S. for patients with and without HCC alike [3, 34]. It is plausible that unabated overweight, obesity, and physical inactivity epidemics in SA are underlying reasons why NASH is undiscriminating in CLD patients with and without HCC. In SA, the prevalence of overweight (70%), obesity (35%), and physical inactivity (35%) are higher in adults, with a high prevalence of T2DM [35–38]. Undoubtedly, NASH is a consequence of this quadruple-prong epidemic (i.e., syndemic) in SA [39]. Other potential consequences can include increases in the number of donors with pre-obesity and obesity, which negatively impacts donor complications and LT outcomes for recipients. A high BMI increases the likelihood of complications in donors [40], and overweight donors are more likely to have hepatic steatosis, which is a predictor of early graft loss [41, 42]. Similarly, the high prevalence of obesity in SA has raised concerns about the possibility of forthcoming reductions in the pool of potential donors in the coming years [43]. A prospective clinical outcome study of 296 children ≤ 14 years of age with severe obesity who underwent sleeve gastrectomy revealed that 65% of them had NASH and 60% had clinically significant fibrosis [44]. These alarming figures further highlight the impact of NAFLD on both future potential recipients and the future pool of potential donors in SA. Furthermore, the persistent metabolic disease in patients with obesity who undergo LT puts them at risk of developing NAFLD post-LT. Additionally, the reversal of the cirrhosis-related catabolic state in addition to the systemic effect of immunosuppressive agents may further worsen the metabolic abnormalities resulting in posttransplant metabolic syndrome and de novo NAFLD [45]. The current study’s findings in concert with this epidemiologic backdrop suggest an urgent need for government-supported public health agencies, healthcare systems, and LT centers to target obesity as a cornerstone for the treatment of NAFLD through innovative health promotion and health education efforts. With the aim to reduce the frequency of sedentary behaviors and obesogenic eating environments as well as improving access to effective obesity treatments (to expand the pool of donors), and especially among those living with CLD (to improve LT outcomes).

Our study had notable limitations and strengths. The study only included a single LT center in SA (the largest and leading liver transplant center in the Middle East), and future iterations of this study are encouraged to include multiple LT centers and a larger cohort of LT recipients. The study was retrospective; thus, it was limited to an analysis of secondary data. All etiologies were not delineated in the study because there were a number of etiologies that were too infrequent (i.e., comprised < 2% of all LTs); and those respective etiologies were grouped together to create a non-specific composite variable (i.e., other etiologies) for statistical analysis. Nonetheless, the number of observational years and the study period were both congruent with the most recent study that published trend analyses of LT indications in the U.S. (e.g., 19 years [2001–2019] and 18 years [2002 to 2019], respectively) [3]. To our knowledge, this was the first study conducted in the Middle East that assessed and compared pre- and post-DAA LT etiology trends among LT recipients.

## Conclusions

Trends in the epidemiology of LT indications among LT recipients in SA have changed. Most notably, NASH has eclipsed HCV in the country. This transposition was likely due to the following happening in tandem: (1) the advent of DAAs for HCV treatment in 2014, and (2) the increasing prevalence of obesity, physical inactivity, and T2DM. In a recently published Markov model, in the general SA population, NAFLD was projected to increase by 48% to 12,534,000 cases by 2030, and consequently, NASH to increase by 96% to 2,688,000 cases in 2030 [46]. Assuredly, projected increases in NAFLD and NASH in the general SA population will likely trickle down to both LT donors and LT candidates. These results now call for an aggressive public health response to minimize and avert future onset of additional clinical and economic strains on health care systems and LT centers in SA.

## Data Availability

The datasets used and/or analyzed during the current study are available from the corresponding author on reasonable request.

## Abbreviations

AIH: Autoimmune hepatitis
ALD: Alcoholic liver disease
APC: Annual percentage changes
BMI: Body mass index
CI: Confidence interval
CLD: Chronic liver disease
CVD: Cardiovascular disease
DAAs: Direct-acting antivirals
ESLD: End-stage liver disease
HBV: Hepatitis B virus
HCV: Hepatitis C virus
HCC: Hepatocellular carcinoma
IRR: Incidence rate ratio
IQR: Interquartile range
KFSHRC: King Faisal Specialist Hospital & Research Centre
LT: Liver transplant
MELD: Model for end-stage liver disease
NAFLD: Nonalcoholic fatty liver disease
NASH: Nonalcoholic steatohepatitis
PSC: Primary sclerosing cholangitis
SA: Saudi Arabia
SCH: Schistosomiasis
SVR: Sustained viral response
T2DM: Type 2 diabetes mellitus
U.S.: United States
VH: Viral hepatitis
WMA: World Medical Association
